# Diagnostic biomarkers for Parkinson’s disease at a glance: where are we?

**DOI:** 10.1007/s00702-018-1910-4

**Published:** 2018-08-25

**Authors:** Ilaria Cova, Alberto Priori

**Affiliations:** 1 Neurology Unit, L. Sacco University Hospital, Milan, Italy; 2 Department of Health Sciences, “Aldo Ravelli” Research Center for Neurotechnology and Experimental Brain Therapeutics, University of Milan and ASST Santi Paolo e Carlo, Milan, Italy

**Keywords:** Parkinson’s disease, Diagnosis, Biomarkers

## Abstract

Parkinson’s disease (PD) is a neurodegenerative disorder whose aetiology remains unclear: degeneration involves several neurotransmission systems, resulting in a heterogeneous disease characterized by motor and non-motor symptoms. PD causes progressive disability that responds only to symptomatic therapies. Future advances include neuroprotective strategies for use in at-risk populations before the clinical onset of disease, hence the continuing need to identify reliable biomarkers that can facilitate the clinical diagnosis of PD. In this evaluative review, we summarize information on potential diagnostic biomarkers for use in the clinical and preclinical stages of PD.

## Introduction

Parkinson’s disease (PD) is the second most common neurodegenerative disorder after Alzheimer’s disease: the incidence rate is 8–18 per 100,000 person-years in prospective population-based studies and the prevalence increases with age, the main known risk factor (Lee and Gilbert 2016). Typical PD symptoms include motor features (bradykinesia, postural disturbances, rigidity or tremor or both) and non-motor features (hyposmia, sleep disorders, autonomic, neuropsychiatric and sensory symptoms). The diagnosis of PD depends mostly on clinical motor findings (Postuma et al. 2015), which appear when half of the substantia nigra (SN) dopamine neurons are lost (Cheng et al. 2010). PD is, therefore, often diagnosed clinically when disease progression is already advanced. Hence the diagnosis is notably inaccurate (from nearly 74% accuracy reached by nonexperts, to 80–84% by movement disorder experts) (Rizzo et al. 2016). Difficulties arise mainly in distinguishing PD from other parkinsonisms. To date, no treatment exists for PD; medications and surgery aim merely to control symptoms for several years. Current treatment strategies focus on dopamine replacement to correct or at least partially correct motor signs caused by dopamine deficiency. The key neuropathology in PD is Lewy body deposition (abnormal aggregates of a misfolded protein called α-synuclein) and consequently neuronal dysfunction, involving many other brain areas and neurotransmitter systems. In their early research, Brack et al. proposed a staging scheme based on rostro-caudal pathological progression (Braak et al. 2003). In this study, they suggested that in the earliest stages, PD damage is confined to non-dopaminergic structures in the lower brainstem, the olfactory bulb or perhaps the peripheral autonomic nervous system, accounting for the early appearance of non-motor symptoms (Chaudhuri et al. 2011). This non-motor theory has disadvantages because it applies mainly to the subset of patients with young onset and long disease duration. Equally controversial is the connection between Lewy pathology and clinical parkinsonian features (Rietdijk et al. 2017).

At present, we lack standardized international criteria supporting PD diagnosis at a preclinical stage: research efforts are, therefore, seeking ways to detect biomarkers for the early diagnosis of PD. Research in recent years, prompted by epidemiological data on risk factors and prodromal biomarkers, has proposed diagnostic criteria based upon the likelihood of prodromal disease (with 80% certainty) but applicable only for research purposes (Berg et al. 2015). Their usefulness consists in defining target populations for eventual future disease-prevention trials with disease-modifying drugs (Mahlknecht et al. 2016).

## Definition of biomarkers

Although the term ‘biomarker’ is often used indiscriminately to describe any change in gene or protein expression the NIH Biomarkers Definitions Working Group defines it more appropriately as “a characteristic that is objectively measured and evaluated as an indicator of normal biological processes, pathogenic processes or pharmacologic responses to a therapeutic intervention” (Biomarkers Definitions Working Group 2001).

Several excellent reports have examined how to use and qualify clinical biomarkers (Freeman et al. 2010). Biomarkers for PD could for example be used to diagnose PD (diagnostic markers), predict the risk of PD or disease progression (prognostic markers), describe disease severity (staging markers) and support treatment choice (theragnostic markers). Diagnostic markers can be useful to recognize PD before motor features become evident or when motor or non-motor signs or both are still insufficient to define disease (prodromal phase) or even to detect an asymptomatic population at risk of PD in whom neurodegeneration is expected to begin (preclinical phase). Diagnostic markers could also help to differentiate PD from other parkinsonian syndromes, insofar as misdiagnosis often takes place early in the disease and diagnostic confirmation needs autopsy reports. The difficulty in identifying early diagnostic criteria for PD depends on the fact that no real biomarker can yet predict illness onset.

In this evaluative review, we will, therefore, summarize the most referred and promising diagnostic biomarkers now under investigation for PD (Tables 1, 2).

**Table 1:** Major diagnostic biomarkers under investigation in Parkinson’s disease

Markers	Diagnostic test statistics	References	Testing cost^a^	Invasiveness	Disadvantages comments
Clinical biomarkers					
Mild motor symptoms	SE 89.3% SP 65.8%	Rizzo et al. (2016)	Low	Low	Late indicators
Levodopa challenge	SE 70.9% SP 81.4%	Merello et al. (2002)	Low	Low	Late indicator
Olfactory dysfunction	SE 60–100% SP 72–94%	Berg et al. (2015)	Low	Low	Lack of standardization in olfactory tests Unclear lead time
REM behaviour sleep disorder	SE 50% SP 40–65% within 10 years	Gagnon et al. (2002) and Postuma et al. (2009)	Low for screens Moderate for PSG	Low	Differences between clinically or PSG-proven
Excessive daytime sleepiness	SE 21–23% SP 87–92	Berg et al. (2015)	Low	Low	Very low predictive positive value (0.5–3.7%)
Constipation	SE 10–50% SP 75–96%	Berg et al. (2015)	Low	Low	Very common in the general population
Depression	SE 0.2–45% SP 75–99.9	Berg et al. (2015)	Low	Low	Very common in the general population
Visual dysfunctions	N/A	Armstrong (2015)	Low	Low	Few studies
Fluid biomarkers	N/A		Moderate or high	Minimally or moderately invasive	Few studies or scarce reproducibility
Tissue biomarkers	Depends on harvesting method	Berg et al. (2015)	Moderate or high	Moderately invasive	Few studies or scarce reproducibility
Imaging biomarkers					
MRI 3 T (7-Tesla)	SE 94.6% (97.7%) SP 94.4% (94.6%)	Mahlknecht et al. (2017)	High	Low	Available only in a research context
DAT imaging	In hyposmic subjects: SE 77.8% SP 94.2% In RBD cohort SE 53.6% SP 98.4%	Iranzo et al. (2010) and Jennings et al. (2014)	High	Minimally invasive	High-risk population bias in main studies Not useful to differentiate between neurodegenerative parkinsonian disorders
Substantia nigra hyperechogenicity	SE 44.4–82.4% SP 82.5–87.2%	Berg et al. (2013) and Iranzo et al. (2014)	Moderate or high	Low	10–20% people have an inadequate bone window
Genetic biomarkers	Depends on gene mutation	Berg et al. (2015)	Moderate	Minimally invasive	Few cases of PD owing to pure monogenetic mutation; different penetrance and age-dependent expressivity
Meta-iodo-benzylguanidine cardiac scintigraphy	SE 90.2% SP 81.9%	Chung and Kim (2015)	Moderate	Minimally invasive	May be negative in genetic Parkinson
Neurophysiological biomarkers	N/A	Priori et al. (2004), Swann et al. (2015) and Wang et al. (2016)	Moderate	Low	Few data on healthy controls
Metabolomic biomarkers	N/A	Chen et al. (2016) and Kori et al. (2016)	Moderate or high	Minimally invasive	Insufficient data
Inflammatory biomarkers	N/A	Chen et al. (2016)	Moderate or high	Minimally or moderately invasive	Insufficient data

*MRI* magnetic resonance imaging, *N/A* not available

^a^Low = visit or questionnaire; moderate = requires a low-cost examination (< 200 US $); high = requires an expensive evaluation (> 200 US $) Postuma and Berg (2016)

**Table 2:** Biomarkers for the prodromal stage, diagnosis and progression of Parkinson’s disease (PD)

Prodromal	Diagnostic	Progression
Clinical Slight MS (parkinsonism) NMS (hyposmia, RBD, EDS, constipation, depression, visual dysfunctions)	Clinical MS (parkinsonism) NMS (hyposmia, RBD, EDS, constipation, depression, visual dysfunctions)	Clinical MS (tremor, postural instability) NMS (hyposmia, cognitive decline, dysautonomia)
Biological fluids Blood (e.g., uric acid)	Biological fluids CSF (α-synuclein species, DJ-1, LRKK2, metabolites) Blood (e.g., uric acid, apolipoprotein A1, LRKK2, inflammation factors) Saliva (α-synuclein species) Urine (biopyrin, LRKK2)	Biological fluids CSF (α-synuclein, A ß oligomers, p-tau) Blood (e.g., uric acid, EGF, IGF-1, BDNF)
Pathology Biopsies (GI tract, salivary glands, olfactory mucosa, skin)	Pathology Microbiota (gut, oral)	–
Imaging Nuclear imaging (nigrostriatal degeneration at DaTscan) Transcranial ultrasound (substantia nigra hyperechogenicity)	Imaging Structural imaging (nigral degeneration on MRI at ≥ 3 T) Functional imaging (remapping of cerebral connectivity at f-MRI) Nuclear imaging (nigrostriatal degeneration at DaTscan, microglial activation at PET imaging, post-ganglionic sympathetic nerve damage on cardiac and salivary gland scintigraphy)	Imaging Functional imaging (hypometabolism patterns on FDG-PET)
–	Neurophysiology Altered ERG, VEP, LFPs	–
–	Omics Proteomics, genomics, lipidomics, glycomics, metabolomics, interactomics	–

*MS* motor symptoms, *NMS* non-motor symptoms, *RBD* REM behaviour sleep disorder, *EDS* excessive daytime sleepiness, *CSF* cerebrospinal fluid, *LRKK2* leucine-rich repeat kinase 2, *EGF* epidermal growth factor, *IGF-1* insulin growth factor, *BDNF* brain-derived neurotrophic factor, *GI* gastrointestinal, *DaTscan* dopamine transporter imaging technique, *MRI* magnetic resonance imaging, *f-MRI* functional-magnetic resonance imaging, *FDG-PET* fluoro-2-deoxy-*d*-glucose positron-emission tomography, *ERG* electroretinogram, *VEP* visual evoked potential, *LFPs* long field potentials

## Clinical signs and symptoms

The most important clinical diagnostic and prognostic markers in PD are still the motor symptoms, crucial also for monitoring the response to symptomatic therapy. The physical examination finding that best correlates with nigrostriatal dopaminergic loss and the core sign of parkinsonism is bradykinesia, associated with rest tremor or rigidity, or both (Postuma et al. 2015). Since Hoen and Yahr first described it in 1967, several studies suggested that tremor is a progression marker for benign PD (Marras et al. 2002). Others underline differences in the clinical course and prognosis between tremor-dominant (TD) and postural instability gait disorder (PIGD) or akinetic-rigid subtypes (Stebbins et al. 2013), who show a more rapid clinical progression and have an increased risk to develop disability and dementia (Rajput et al. 2009). Motor impairment is routinely assessed with a clinical rating scale, such as the Unified Parkinson’s disease rating scale (UPDRS) or revised version of the Movement Disorder Society (MDS-UPDRS). The disease-modifying efficacy of a therapy that also provides a symptomatic effect is troublesome to detect. An acute levodopa challenge supports the clinical diagnosis of PD with a specificity that seems to increase in patients with mild symptoms (Merello et al. 2002).

Subthreshold parkinsonism detected by experts in PD assessment (i.e., UPDRS > 3 excluding tremor action) is considered as a marker of prodromal disease that is unlikely to progress to manifest PD (Berg et al. 2015).

Because PD measures are subjective and rater-dependent, two decades ago researchers began to consider using objective monitoring systems, which lead to the development of wearable technology (such as watches, bracelets) and connected devices (such as smartphone apps). These tools gather valuable early diagnostic data on kinematic variables in a non-clinical setting, potentially enhancing clinical diagnosis and care (Del Din et al. 2016; Zhan et al. 2018).

**Fig. 1:**
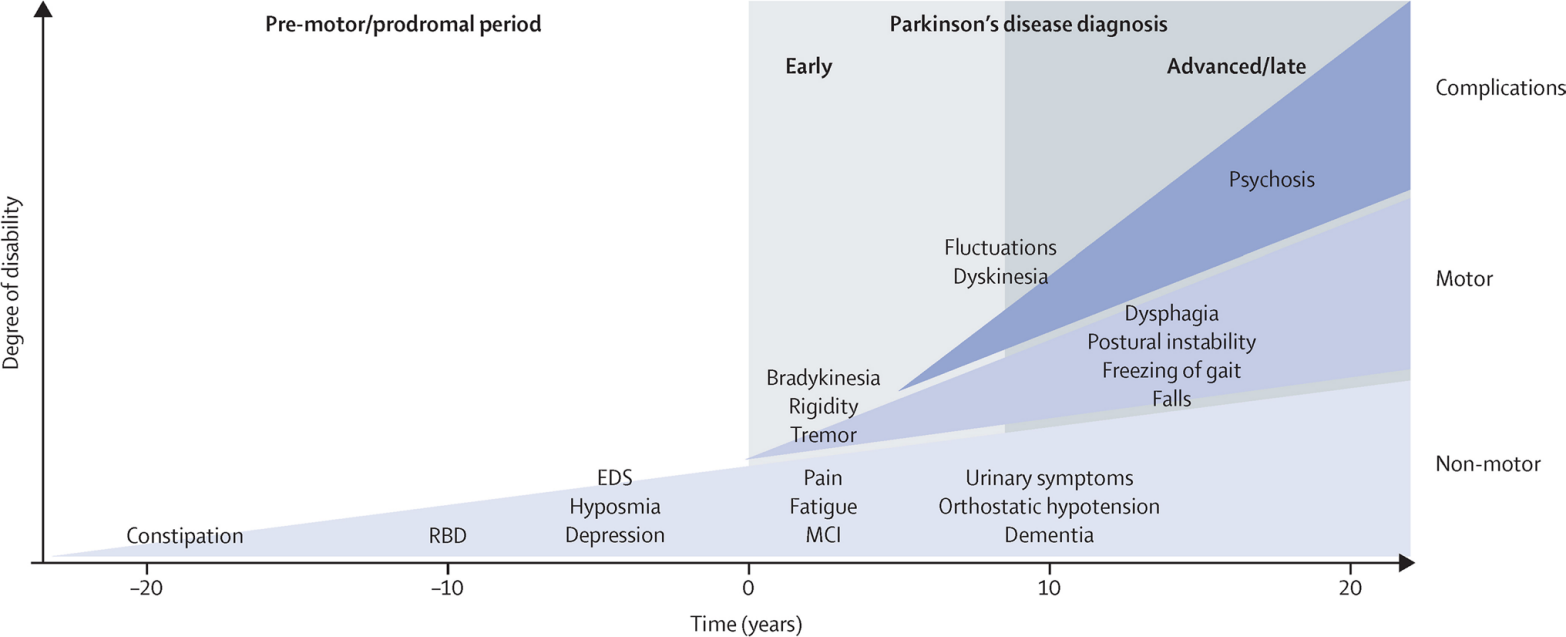
Parkinson’s disease progression. Non-motor symptoms (NMS) may emerge in a pre-motor or prodromal stage reflecting degeneration in extra-nigral areas before the loss of nigral dopamine neurons. Conversely, the motor symptoms currently required for diagnosis manifest later. From Kalia and Lang (10.1016/S0140-6736(14)61393-3) 2015;386:896–912, copyright (2015) with permission from Elsevier

Besides motor features, some non-motor symptoms (NMS) appear specific for PD as well as for other synucleinopathies, neurodegenerative diseases characterized by the abnormal accumulation of α-synuclein aggregates in the nervous system (Lewy body dementia and multiple system atrophy). NMS may appear in a prodromal disease stage (Chaudhuri and Schapira 2009). Early NMS reflect degeneration in extra-nigral areas before the loss of nigral dopamine neurons and include olfactory dysfunction, REM sleep behaviour disorder, autonomic dysfunction (such as constipation), and depression (Berg et al. 2013) (Fig. 1).

### Olfactory dysfunction

The olfactory system is one of the earliest structures affected in PD and hyposmia is one of the NMS in PD (Xiao et al. 2014). The “olfactory vector hypothesis” supports the idea that the olfactory pathway could be an entry-point for certain pathogens with a putative role in starting the disease (Doty 2008). Given that this system’s particular anatomy enables it to bypass the blood–brain barrier, some have also suggested that PD might be a primary olfactory disorder (Hawkes et al. 1999; Doty 2008). Some findings nevertheless excluded neurodegeneration involving dopamine neurons in the olfactory bulb in patients with PD; some evidence even indicates a paradoxical increase in dopamine neurons in some PD patients compared with controls (Duda 2010). Not all patients with PD experience olfactory problems, although sensitivity rates range from 45% up to 96% (Haehner et al. 2009). These differences may reflect the type, or the combination of olfactory tests used or both, and their normative data. Equally important, the age distribution of PD and controls varies between different investigations. Nor can we ignore that hyposmia is a common phenomenon during aging (Welge-Lüssen 2009): smell dysfunction affects more than 50% of people aged 65 years and over and about 75% of subjects older than 80 years (Doty et al. 1984). Hyposmia can also be the early expression of cognitive disorders: some evidence also describes severe olfactory deficits as a predictive feature of PD with dementia (PDD) (Takeda et al. 2014). Subjective olfactory impairment (through information from history-taking or self-reported olfactory questionnaires) may not overlap with an objective injury and a loss of awareness of hyposmia may be associated with early cognitive impairment in PD (Kawasaki et al. 2016). Smell is less impaired, however, in other common causes of parkinsonism (Wenning et al. 1995), in synucleinopathies such as multiple system atrophy (MSA), and in tauopathies such as corticobasal degeneration (CBD) and progressive supranuclear palsy (PSP); an olfactory dysfunction is infrequent also in essential tremor (Busenbark et al. 1992). Other studies found that PD patients with predominant PIGD lose their sense of smell more often than those with TD (Stern et al. 1994). In conclusion, hyposmia can be useful as a preclinical marker of disease in combination with other biomarkers to increase the predictive value but not alone. For example, combined assessment of hyposmia, motor asymmetry and a typical finding at ultrasound (midbrain hyper-echogenicity) could improve diagnostic accuracy in early PD (Poewe and Mahlknecht 2012).

### REM behaviour sleep disorder

Another early PD feature indicating brainstem involvement, supported by the finding of Lewy body pathology, is rapid eye movement (REM) sleep behaviour disorder (Boeve et al. 2007). REM sleep behaviour disorder (RBD) is a parasomnia which involves abnormal behaviour caused by a loss of the physiological motor inhibition (usually present during REM sleep); this disorder leads to varying degrees of complex motor activity which ranges from sleep talking to violent dream enacting behaviours potentially harmful for the patient or bed partner. The reference standard to confirm RBD is a polysomnogram (PSG). One way to avoid this resource-demanding procedure is to interview the patient’s partner with specific questionnaires (Al-Qassabi et al. 2017). RBD affects 0.38–2.01% of people, but its prevalence is higher in synucleinopathies (Jiang et al. 2016) and is an early NMS that appears decades before motor involvement not only in PD, but also in dementia with Lewy bodies (DLB), or MSA (Claassen et al. 2010). The presence of RBD increases the risk of these neurodegenerative disorders developing in up to 40–65% of patients at 10 years (Postuma et al. 2009) and is the prodromal marker with the highest positive likelihood ratio (LR): patients with positive PSG are 130 times more likely than those without to have PD. Positive LRs dramatically decrease when RBD is assessed by means of screen questionnaire even with > 80% specificity, from 130 to 2.2 (Postuma et al. 2015). Despite its high specificity, RBD is present in less than half of all cases of PD (Gagnon et al. 2002), so it is an early sign with scarce sensitivity. In summary, RBD alone is unreliable as a diagnostic biomarker. Combining the presence of RBD as a prodromic NMS with other markers could further increase its predictive value.

### Excessive daytime sleepiness

Excessive daytime sleepiness (EDS) consists in the inability to maintain wakefulness during the day, with sleep occurring unintentionally or at inappropriate times. EDS is among the most common sleep-related patient symptoms, affecting an estimated 20% of the population (Pagel 2009). It can manifest as primary hypersomnia of central origin or more commonly secondary to sleep disorders (for example, sleep deprivation and obstructive sleep apnoea), to medication effects or to other medical and psychiatric conditions.

EDS is a common feature in PD and can affect up to 50% of patients taking dopamine agonist medication (Hobson et al. 2002). A prospective follow-up study of incident clinical PD in men has shown that the presence of EDS increased the risk of disease up to threefold (Abbott et al. 2005). EDS could also appear during the course of the disease; besides the dopamine agonist dose, disparate factors such as male gender, poorer night-time sleep, cognitive impairment, hallucinations and autonomic dysfunction, have been associated with higher EDS scores over time (Zhu et al. 2016). In two population-based studies, EDS showed a positive LR 2.2 to predict PD, so despite its low VPP it can be considered a prodromal marker (Berg et al. 2015).

### Insomnia

Insomnia is defined as the difficulty to initiate or maintain sleep or the presence of early awakenings or both problems. It is the most common sleep disturbance in PD patients, manifesting in up to 80% of cases, and is multifactorial (due to neurodegeneration, depression, anxiety, motor fluctuations, PD drugs and their withdrawal) (Al-Qassabi et al. 2017). Insomnia may arise at any PD stage, but its prevalence seems to increase along with the duration (Loddo et al. 2017). Too little evidence is available to justify including insomnia as a prodromal marker of PD.

### Constipation

A considerably more sensitive early NMS in PD is constipation (Berg et al. 2013), which affects up to 80% of PD patients (Noyce et al. 2012). Despite its sensitivity, this symptom has a low specificity because it is relatively frequent also in the general population and its prevalence increases with age (Higgins and Johanson 2004). A population study (Savica et al. 2009) suggested that constipation arising also more than 20 years before motor symptom onset is associated with an increased risk of PD. The various published studies fail to define constipation in a uniform manner. A review published in recent years (Knudsen et al. 2017) has identified 12 different criteria. Among these, the ROME criteria appear the most valid tool for investigating colonic and anorectal dysfunctions (Drossman and Dumitrascu 2006). These findings notwithstanding, idiopathic constipation is one of the strongest risk factors for PD: men reporting a bowel movement frequency of < 1 per day showed an odds ratio (OR) for PD of 2.3 compared with those reporting 1 per day (Abbott et al. 2001).

Colorectal transit time (CTT) is prolonged in approximately 80% of untreated de novo PD patients, but appears uncorrelated with subjective constipation symptoms (Knudsen et al. 2017). Hence, CTT studied with radio opaque markers may be a potential biomarker for prodromal PD.

Α-synuclein accumulation and other neurodegenerative changes in the enteric nervous system, in addition to signs of local inflammation, oxidative stress and increased mucosal permeability, are associated with prolonged CTT and constipation in the earliest stages of PD (Scheperjans et al. 2015). These features, supporting the caudal-rostral progression of α-synuclein pathology suggested by Braak et al. (2003), led some to hypothesize that such environmental factors could act primarily through the gut, implying that gut microbiota might play a mediatory role. Subsequent research discovering these gut alterations in PD is only the tip of the iceberg. A promising approach for detecting α-synuclein pathology could be to take gastrointestinal tract biopsies (Cersosimo 2015) thus providing a new, although invasive, diagnostic biomarker for PD.

### Depression

About 35% of patients with PD suffer from severe depression (Reijnders et al. 2008). Given that depressive symptoms may precede the onset of motor symptoms in 30% of cases and their incidence increases during the few years preceding the diagnosis, depression has a potential role in diagnosing pre-motor PD. Depression in PD seems related to multiple neurotransmitter dysfunction, involving not only dopamine in the substantia nigra, but also serotonin in the raphe nuclei and noradrenaline in the locus coeruleus (Borgonovo et al. 2017). Like constipation, owing to its high prevalence in the general population, depression is not specific as a stand-alone biomarker of prodromal PD, but can coexist with other potential markers such as family history and substantia nigra hyperechogenicity (Liepelt-Scarfone et al. 2011). Depression is also tricky to diagnose in a patient with clinically manifest PD because it overlaps with motor symptoms, such as slowing and bradyphrenia and with other NMS, for example sleep and appetite disturbance, loss of concentration and interest and impaired libido. Patients with PD can maintain intact affective responses but may have difficulties in translating such feelings into motor phenomena (Rickards 2005). A disorder often mistaken for depression in PD is abulia. Abulia can bias the estimated prevalence rates for a depressive syndrome in PD. Abulia is a disorder of diminished motivation due to damaged connections between the anterior cingulate area and striatum, so that abulic patients have no desire to do things without getting bored (Heimer et al. 2008). Some help in differentiating depression from abulia comes from appropriate diagnostic tools (Torbey et al. 2015).

### Cognition

Cognitive impairment in PD is due to a fronto-striatal syndrome which mainly affects executive function in milder disease stages; a widespread failure in neurotransmitter systems besides the dopaminergic one, such as noradrenergic, serotoninergic and cholinergic systems, is more evident with disease progression. Though cognitive decline is a well-known NMS in the late stages of PD, increasing in parallel with age and PD duration, mild cognitive impairment is recognized also in 15–20% of patients in the early PD stages; approximately 80% of patients have dementia during the disease course (O’Callaghan and Lewis 2017). Cognition cannot be considered as a pre-motor biomarker, but it has a key role as both a staging and prognostic biomarker, as well as in sub-typing and disease stratification. An increase incidence of cognitive impairment is evident in the non-tremor, akinetic-rigid phenotype. Executive dysfunction has been associated with a more severe gait dysfunction, freezing of gait and postural instability, whereas specific cognitive domains were differentially related to different PIGD components (i.e., visual impairment to a more severe freezing of gait, memory impairment to a worse postural instability) (Kelly et al. 2015). The NMS most associated with cognitive decline are early visual hallucinations, RBD, hyposmia and depression. Dementia associated with at least one feature among fluctuating cognition, visual hallucinations, RBD and parkinsonism allow a diagnosis of DLB. No major clinical differences exist between DLB and PDD, both of which belong both to the Lewy body dementia. The DLB Consortium has developed an arbitrary diagnostic distinction (McKeith et al. 2017) based on the temporal appearance of motor and cognitive symptoms (the “1 year-rule” states that cognitive decline manifests before or within a year after the onset of parkinsonism in DLB).

### Visual dysfunction

Among other NMS, PD patients may have various visual disturbances that can be sensitive markers of disease, including changes in colour vision and contrast sensitivity possibly due to a loss of dopaminergic amacrine retinal cells, and difficulties with complex visual tasks (for example mental rotation and emotion recognition) caused by a cortical visuoperceptual dysfunction (Weil et al. 2016). In a study conducted in 2010, Diederich et al. (2010) noted that deficits in colour and contrast sensitivity seem to discriminate early diagnosis of PD within 3 years better than other NMS do. Visuo-perceptual deficits rarely arise in other parkinsonisms and could serve to distinguish idiopathic PD from other diseases. Another visual dysfunction reported in PD is a decrease in retinal nerve-fibre layer thickness on optical coherence tomography (Moschos et al. 2011). Some evidence also suggests as a retinal biomarker a mathematical model quantifying foveal symmetry and breadth (Slotnick et al. 2015). More data are needed on the prevalence of visual markers in the preclinical stage of PD to allow their future use as biomarkers.

## Biological fluids

Altered α-synuclein metabolism in the central nervous system (CNS) has a central role in the pathogenesis of PD and may manifest also in the periphery. Studies conducted in recent years have focused on determining α-synuclein species in different fluids and tissues. Although α-synuclein is mainly expressed by neuronal cells as a cytoplasmic protein in its native form or in aggregated pathological (oligomeric, phosphorylated) forms (Abd-Elhadi et al. 2016), because it also has access to the extracellular space it can be detected in cerebrospinal fluid (CSF). Because observations in recent years have linked CSF α-synuclein levels to PD, some have speculated that they could reflect disease severity (Hall et al. 2015), though others disagree (van Dijk et al. 2014). CSF α-synuclein levels seem to decrease with age also in healthy subjects (Koehler et al. 2015). Total α-synuclein is decreased in synucleinopathies, but is not specific for PD, whereas data for aggregated forms of α-synuclein yield controversial results (Andersen et al. 2016). Phosphorylated α-synuclein combined with total α-synuclein concentrations in CSF may help in distinguishing PD from atypical parkinsonisms (MSA, PSP) (Wang et al. 2012), but further studies are needed in independent cohorts of patients.

Other major potential CSF biomarkers studied include DJ-1, whose mutations are a rare cause of PD (Hong et al. 2010), Aβ42, which seems to correlate with cognitive impairment, and different forms of tau and neurofilament light chains that might help to differentiate PD from other α-synucleinopathies (such as MSA) and primary tauopathies (PSP and CBD) (Magdalinou et al. 2014). Despite these encouraging findings, future research needs to seek more data. Owing to the degeneration in catecholaminergic neurons, some report lower levels of dopamine metabolites, such as dihydroxyphenyl acetate (DOPAC) and homovanillic acid (HVA) and noradrenergic metabolites including dihydroxyphenylglycol (DHPG) and 3-methoxy-4-hydroxyphenylglycol (MHPG) in CSF from patients with PD than from controls (Lee et al. 2017). In a case–control study (217 vs. 26 subjects) that lacked healthy elderly controls, others proposed as a possible state and trait biomarker for PD, the ratio of the purine metabolite xanthine over HVA (LeWitt et al. 2011).

Because lumbar puncture is an invasive procedure, and increasing evidence suggests that α-synuclein passes through the blood–brain barrier, recent efforts concentrate on assaying it in plasma rather than CSF. This research has yielded ambiguous results owing to the various assay techniques, the different protein forms assayed (total, oligomeric and phosphorylated) and the small populations enrolled (El-Agnaf et al. 2006; Lee et al. 2006; Li et al. 2007; Duran et al. 2010; Shi et al. 2010; Foulds et al. 2013); others also suggested gender-related differences in plasma α-synuclein expression in PD (Caranci et al. 2013). Blood has been a target also for assaying DJ-1, a protein involved in the pathogenesis of PD through oxidative stress and mitochondrial dysfunction. Assaying total DJ-1 levels in PD patients has produced controversial results (Hong et al. 2010; Shi et al. 2010), whereas fractions of specific DJ-1 isoforms differed in PD patients and healthy controls. Although some researchers suggested high oxidized DJ-1 protein levels in red blood cells as a potential biomarker for early PD, the study enrolled a small sample (Ogawa et al. 2014). Fractions of specific isoforms of 4-hydroxy-2-nonenal-modified DJ-1 differed significantly in patients with PD and controls (Lin et al. 2012) and within stages of PD, suggesting a potential as a staging biological marker of disease. These findings merit confirmation in further studies.

Another blood biomarker is serum urate (one of the major anti-oxidants in humans), levels of which have been known for 20 years to be lower in PD than in healthy persons (Davis et al. 1996). Urate has been proposed as a staging marker in the PRECEPT cohort (Schwarzschild et al. 2008): clinical progression was slower in a cohort of 399 de novo PD men (but not in women) with the highest serum urate level than in men with the lowest levels.

Other unbiased blood-based biomarkers for PD include epidermal growth factor, whose level correlates with cognitive performance in PD (Chen-Plotkin et al. 2011) and apolipoprotein A1, which seems to decrease the risk of developing the disease. Apolipoprotein-1 negatively correlates with dopaminergic denervation in subjects with hyposmia and a family history of PD (Qiang et al. 2013) and its level has a maximum sensitivity of 71% and specificity of 60% in differentiating patients with PD from healthy controls (Swanson et al. 2015).

Another potentially readily accessible body fluid is saliva; two studies reported a reduction in total α-synuclein levels and elevation in oligomeric α-synuclein and DJ-1 in the saliva of PD patients compared with controls (Masters et al. 2015; Vivacqua et al. 2016), confirming previously reported results (Stewart et al. 2014).

The search for fluid PD biomarkers focusses also on the urine, as in the BIOFIND study (Kang et al. 2016). In a case–control study (92 patients vs. 65 healthy controls) others found elevated excretion of urinary biopyrin, an oxidative metabolite of bilirubin (Luan et al. 2015), but this promising finding remains unreplicated.

## Pathology

**Fig. 2:**
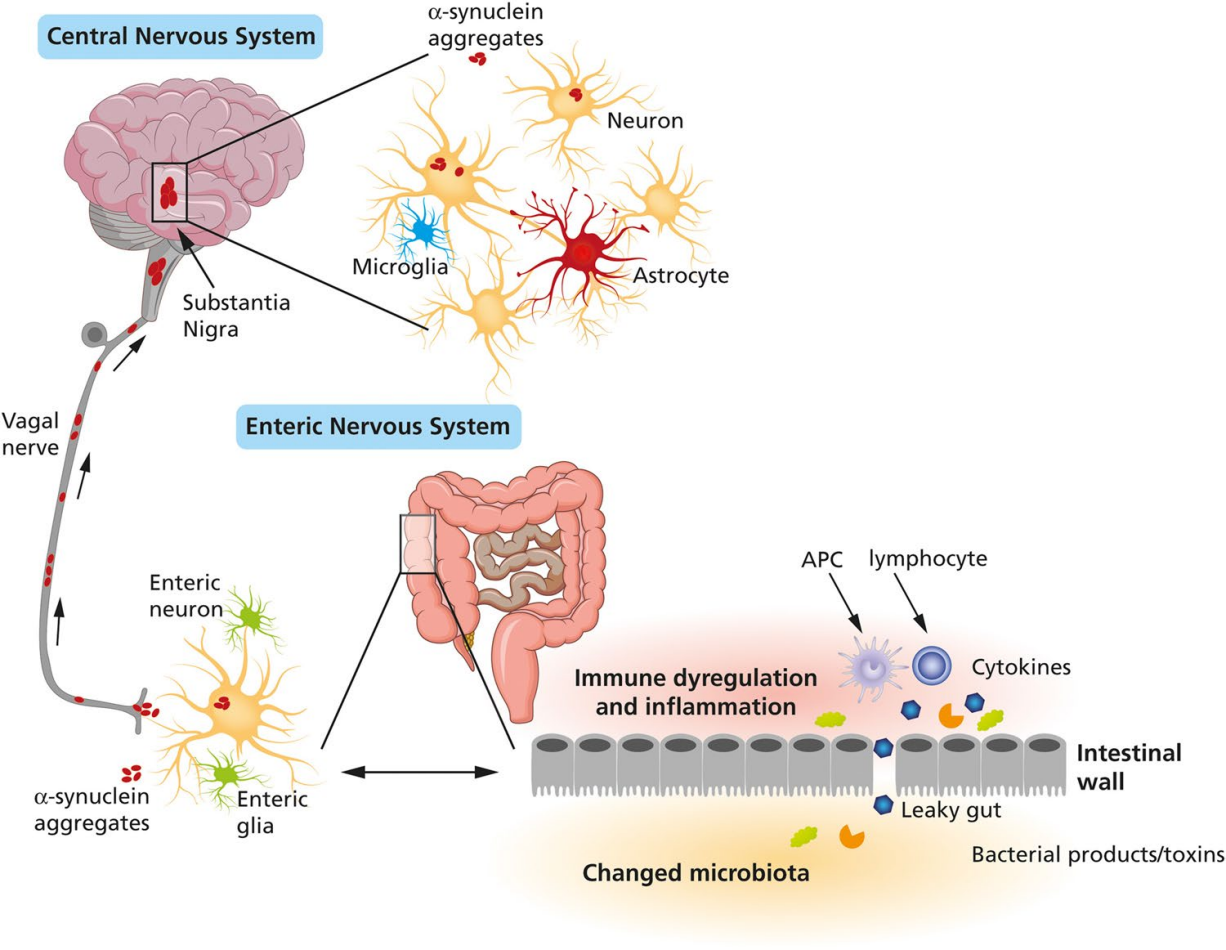
An abnormal microbiota in PD (in favour of proinflammatory species) can lead to immune dysregulation and intestinal nervous system inflammation and consequently to a dysfunctional intestinal barrier: α-synuclein pathology can thus spread through the vagal nerve from peripheral autonomic neurons in the gastrointestinal apparatus to the central nervous system. From Perez-Pardo (10.1016/j.ejphar.2017.05.042) 2017 with permission from Elsevier

Given the known presence of extr-anigral PD pathology, research efforts have focused on demonstrating Lewy type α-synuclein deposition in peripheral tissues, especially in olfactory bulb, gastrointestinal tract (GI) (such as submandibular gland, oesophagus, stomach and colon) and skin (Lee et al. 2017). Yet tissue biopsies are unlikely to help distinguish PD from other synucleinopathies (Mehta and Adler 2016). A review published in recent years (Schneider et al. 2016) evaluated the sensitivity and specificity of total and phosphorylated α-synuclein detected in biopsies from various tissues from PD patients, despite the difficulties in analysing the many disparate harvesting methods (such as vivo vs. post-mortem samples) used in the various studies. Tissues obtained from the GI tract and salivary glands showed a better sensitivity and specificity for total α-synuclein than did skin and or olfactory mucosa or bulb, the most promising tissue biomarker for PD, whereas phosphorylated α-synuclein resulted inadequate. This finding is not surprising given that the gut could be a starting point for PD through which the disease may spread along the brain–gut axis (Mulak and Bonaz 2015). Some observations imply that α-synuclein pathology in peripheral autonomic neurons in the gastrointestinal apparatus may precede CNS pathology (Greene 2014). Another potential gut biomarker emerges from studies investigating faecal microbiome (brain-gut-microbiota axis), changes in whose complex equilibrium can be linked to the development of several diseases, including PD. Nine studies over recent years have suggested an intestinal dysbiosis in PD due to an imbalance in the bacterial strains in favour of pro-inflammatory species, (Scheperjans 2017). Indeed, GI dysfunction manifests in over 80% of PD subjects. An inflammatory condition, associated with a reduction in intestinal cell adhesion (Clairembault et al. 2015), may result in a dysfunction involving the intestinal barrier (leaky gut syndrome). These changes may be the gateway allowing synuclein pathology to spread through the enteric nervous system toward the CNS, as the Braak hypothesis suggested (Braak et al. 2003); as a possible mechanism, others have postulated prion-like propagation (Chauhan and Jeans 2015) (Fig. 2). All the studies published until now have shown significant differences between the microbiota in established PD and controls favouring proinflammatory species in the patients’ group (Tremlett et al. 2017). Combined with other measures, these distinctions may help in developing clinically useful microbial biomarkers for PD. Longitudinal studies starting in the premotor phase would help to investigate the causality of such alterations. Evidence obtained in recent years underlines that alterations in gut microbiome precede the motor symptoms of PD, given that they were found also in RBD (Heintz-Buschart et al. 2017), the NMS associated with the major likelihood to progress to a synucleinopathy.

Oral microbiota seems to differ in bacterial taxa from patients with PD compared with controls, whereas nasal microbiota were similar in two studies (Pereira et al. 2017; Heintz-Buschart et al. 2017). Last, some evidence supports assaying α-synuclein deposition in cutaneous autonomic nerves (Wang et al. 2013): whether tissue α-synuclein might be a marker for disease progression awaits evidence from longitudinal studies.

## Imaging

**Fig. 3:**
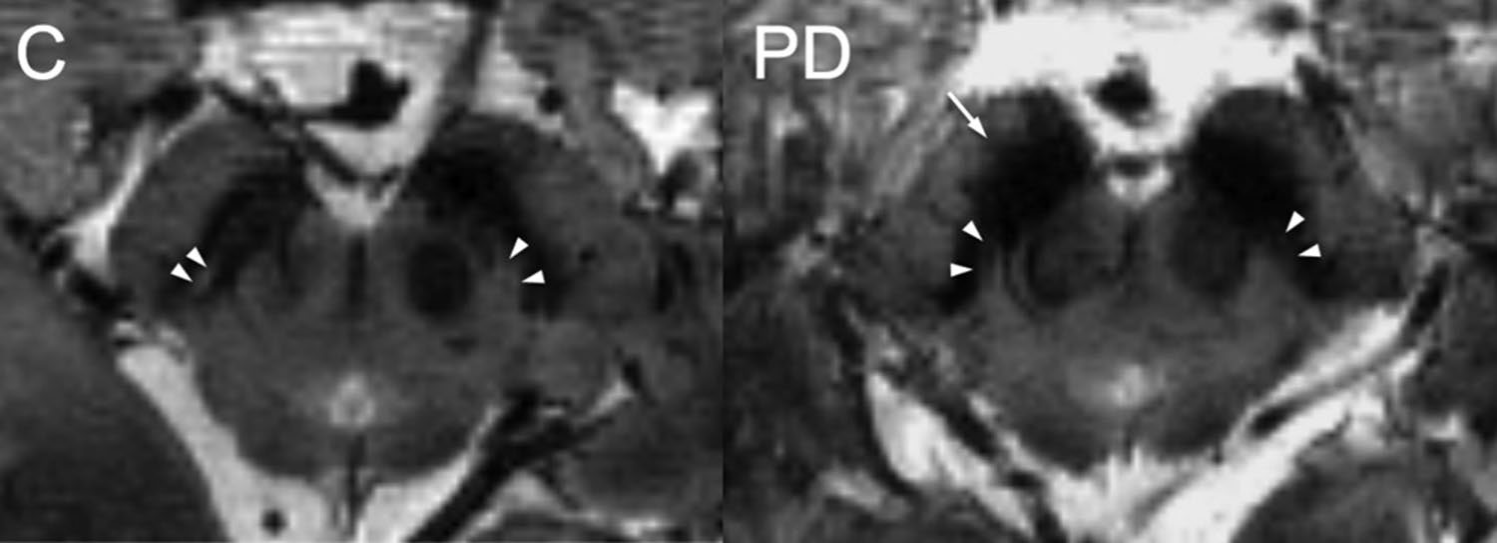
7-Tesla magnetic resonance imaging scan (MRI) scan showing mesencephalic anatomy in a control (C) and a patient with Parkinson’s disease (PD). Nigrosome-1 (white arrowheads) appears as a hyperintense pocket (due to a low iron content) in the control subject whereas it is not visible in the PD patient, in whom shape analysis shows undulation in the anterolateral perimeter of the subthalamic nucleus (arrow). From Lehéricy et al. (10.1002/mds.26043), 2014 with permission from John Wiley and Sons

Progressive dopaminergic cell loss in the pars compacta (SNpc) is the hallmark of PD and has been studied in detail histologically (Jellinger 2012). Unlike conventional magnetic resonance imaging (MRI) acquired with a 1 or 1.5 T scanner, ultra-high field 7 T (7 T) MRI, by providing better spatial resolution and contrast, can now detect typical PD-related changes in SNpc morphology (Lehéricy et al. 2014) (Fig. 3). Neuropathologically, dopamine-containing neurons are distributed in calbindin-poor zones (nigrosomes) and in the calbindin-positive region (matrix). Nigrosome-1 indicates the area of maximal dopamine depletion and is considered the most sensitive pathological marker of neurodegeneration in PD (Damier et al. 1999). 7 T MRI displays a nigral hyperintensity overlapping the neuropathological characterization of nigrosome 1 and is useful to distinguish patients with PD from controls owing to a loss of hyperintensity areas on T2*-weighted images, present also in patients with MSA with predominant parkinsonism and PSP (Kim et al. 2016). This marker could reflect the loss of melanized neurons and the increase in iron deposition in the SNc (Jellinger 2012). A metanalysis (including 10 studies) conducted in recent years has established that hyperintensity on iron-sensitive dorsolateral nigral MRI sequences can provide excellent diagnostic accuracy. Its absence demonstrated an overall sensitivity and specificity of 97.7 and 94.6% (3 and 7 T) and of 94.6 and 94.4% (3 T only) for PD vs. controls, thus providing a marker for nigral pathology and for a neurodegenerative form of parkinsonian disorders (Mahlknecht et al. 2017).

Because the shape of the degenerated nigrosome-1 and its surrounding structures in axial imaging on 3 T susceptibility-weighted MRI in PD has been compared to “a swallow tail”, this imaging sign has been proposed as a potential diagnostic tool for nigral degeneration in PD (Schwarz et al. 2014; Gao et al. 2016).

As tools for studying the pathophysiology of PD in vivo, functional neuroimaging techniques (fMRI) have promising potential. Various methods for analysing data for subjects in the resting state (awake without performing any particular task) have been applied to detect functional connectivity abnormalities in PD. Dopamine depletion causes a cerebral connectivity remapping across the whole brain (better identified through “network-based” and “graph analysis” techniques), between specific regions (as shown by “seed-based” and “effective connectivity” methods) and regional anomalies (detected by analysing “regional homogeneity” and “amplitude of low-frequency fluctuations”) (Tahmasian et al. 2015). Drawbacks currently limiting resting-state fMRI as a biomarker for PD include heterogeneous methods and insufficient published works using similar methods.

Ample information on dopaminergic denervation comes from studies using radiotracer imaging in target structures (striatum). These imaging techniques are currently used in research and clinical settings to improve the diagnostic accuracy for PD in positron emission tomography (PET) and in single-photon emission CT (SPECT). The most commonly used radioligands are substances contained in presynaptic terminals, such as dopa decarboxylase (18F-fluorodopa PET), vesicular monoamine transporter type 2 (11C-dihydrotetrabenazine) and dopamine transporter (DAT) (123I-β-CIT SPECT and 99mTcTRODAT-1). DAT density has been used as an imaging biomarker for diagnosing PD since 2011, because decreased DAT uptake, expressing nigrostriatal dopamine neuron loss, is evident before motor features of PD appear. Even if DaTSCAN is more sensitive than clinical observations, it lacks diagnostic specificity and cannot distinguish PD from other synucleinopathies (MSA, DLB) or tauopathies (PSP) nor measure disease progression (Perlmutter and Norris 2014). What research needs to do now is to develop new tracers based on molecular imaging advances thus providing in vivo markers for detecting the underlying pathology in PD (Strafella et al. 2017).

Over 90% of PD patients show SN hyperechogenicity assessed by transcranial ultrasound compared with 10% of controls. Given that this echo-feature remains stable during disease progression, it could act as a vulnerability factor for PD during patients’ lifetime (Behnke et al. 2010). Because SN hyperechogenicity is rarely present in degenerative parkinsonisms, it differentiates PD from MSA and PSP (Pilotto et al. 2015). Like every ultrasound technique, transcranial ultrasound is operator-dependent and in up to 10–20% of people is also limited by a poor temporal bone window (Behnke et al. 2010).

Meta-iodobenzylguanidine (MIBG) cardiac scintigraphy is a technique used to evaluate post-ganglionic presynaptic cardiac sympathetic nerves in heart diseases through a false neurotransmitter analogous to norepinephrine. Given the early sympathetic nerve degeneration during PD, reduced cardiac MIBG uptake is a specific marker for Lewy bodies in the autonomic nervous system (Lucio et al. 2013). This imaging technique is also useful for differentiating PD from MSA, a disease that spares postganglionic sympathetic nerves, but pathologically damages preganglionic neurons. The MIBG technique can also discriminate between PSP and DLB (Lucio et al. 2013). MIBG uptake in the parotid and submandibular glands is significantly lower in PD patients than in controls (Haqparwar et al. 2016).

## Genetics

PD has a multifactorial aetiology including lifestyle, environment and genetics and, although only about 10% of patients report a positive family history, at least 30% of the risk of developing this disorder depends directly on genetic factors. Genome-wide association studies have identified at least 28 genetic risk loci associated with PD (Taymans et al. 2017). Few cases of PD are determined by a pure monogenic mutation, and also in these patients, PD development cannot be taken for granted given the possible low penetrance and age-dependent variable expressivity. Age at onset remains highly variable even in some carriers of high-penetrance mutations (Gasser 2009), such as the α-synuclein gene (SNCA) mutation, recognized as a rare cause of autosomal dominantly inherited PD. Leucine-rich repeat kinase 2 (LRRK2) is another gene of particular interest because it induces pleomorphic effects, shows high phenotypic variability among subjects, is also involved in sporadic disease forms, and is the most common genetic cause of PD in the world (Taymans et al. 2017). LRKK2 could be assayed in tissues and biofluids relevant to disease and its expression and phosphorylation levels could have potential as a PD-related biomarker (Taymans et al. 2017).

**Fig. 4:**
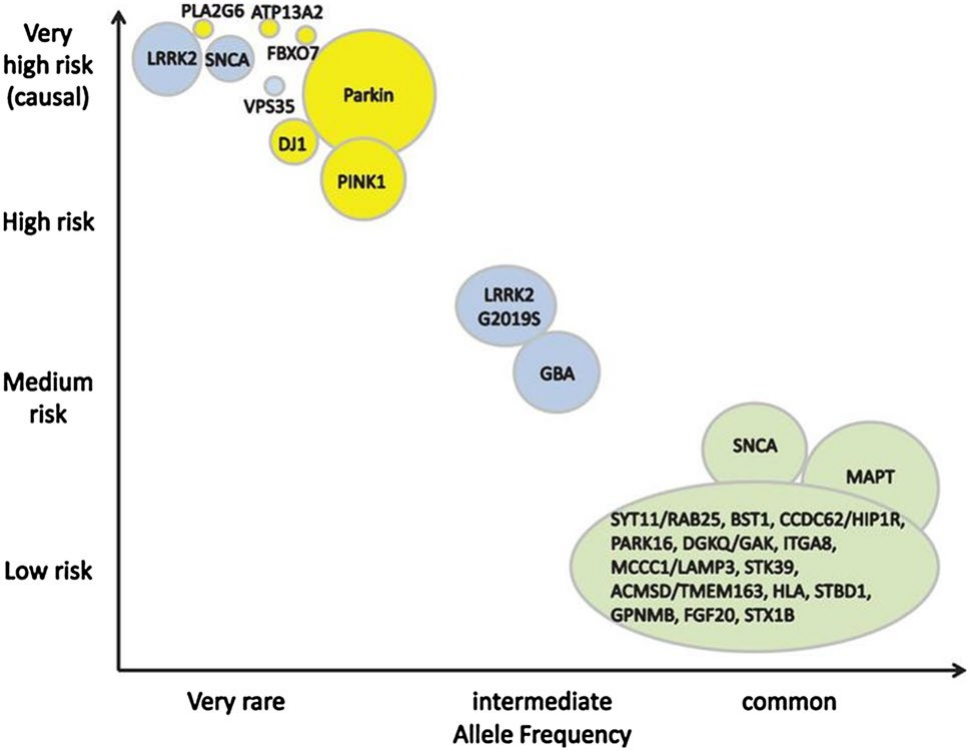
Genetic risk factors for Parkinson’s disease. Parkinson’s disease (PD) has a multifactorial genesis and, although only about 10% of patients have a family history, at least 30% of the risk of PD developing depends on genetic factors, which differ in strength and allele frequencies. The size of the bubbles corresponds to population; the size of the rings corresponds to allele frequencies; inheritance is shown by colours (blue = dominant, yellow = recessive, green = risk loci). From Gasser (10.3233/JPD-140507) 2015 with permission of IOS Press

Some monogenic and all sporadic forms of PD share the same pathological mechanism, namely intraneuronal accumulation of α-synuclein aggregates in Lewy bodies and Lewy neuritis in the SNpc and striatum. Mutated genes contribute to the pathogenesis of PD through various mechanisms (some still unknown): some by producing misfolded proteins (α-synuclein gene duplications, triplications and point mutations), others due to dysregulated mitochondrial homeostasis (Parkin, DJ1, PINK1 and FBXO7 mutations) or to altered lysosomal degradation (ATP13A2 and GBA mutations). Other possible candidate biomarkers for PD include the aforementioned misfolded proteins, e.g., SNCA gene-encoded α-synuclein (Miller and O’Callaghan 2015) (Fig. 4).

## Neurophysiological biomarkers

In neurophysiological studies conducted in the 1980s, researchers describe defects in visual processing involving dopamine neurons including altered electroretinogram (ERG) waves in PD patients: for example, reduced ‘b’ wave amplitude at flash ERG, a reduced P50 amplitude and a delayed latency at pattern ERG. Visual evoked potentials in response to coloured stimuli are also affected in PD; some patient’s visual evoked potentials also show a decreased amplitude along with an increased latency for all chromatic stimuli, above all for blue–yellow horizontal gratings, as well as an increased P100 latency to a checkboard stimulus. This last finding outlined a delay in visual processing at a certain visual system level, maybe involving the cholinergic system (Armstrong 2015). No current evidence suggests that these neurophysiological biomarkers will accelerate the diagnosis of PD.

Subthalamic nucleus local field potentials recorded from electrodes implanted intraoperatively in the basal ganglia disclosed oscillations in the β frequency range, the range that levodopa and deep brain stimulation (DBS) decrease while motor symptoms improve (Priori et al. 2004). Some evidence suggests that this β activity, as well as β phase coupling to high-frequency oscillation amplitudes is a specific biomarker for the parkinsonian condition, (Wang et al. 2016), but few studies have investigated these patterns in healthy controls (Swann et al. 2015).

## Omics

During continued research into PD, the study of disease networks and molecular pathways through a bioinformatics approach provides new potential ‘Omics’ biomarkers.

Omics testing can be applied to various biological media (tissue, CSF, blood, urine) and aims to analyse functional molecules such as proteins (proteomics), DNA/RNA (genomics), lipids (lipidomics), carbohydrates (glycomics) and metabolites (metabolomics), interactions between molecules (interactomics), potentially involved in pathways that are associated with dopaminergic neurodegeneration and subsequently PD (Redenšek et al. 2018).

Metabolomics refers to the study of quantitative data on cellular processes, molecular connections and metabolic pathways appearing in complex diseases such as neurodegenerative disorders. A recent review (Kori et al. 2016) reported that to date 54 metabolites have been studied as biomarkers for PD. Some of these metabolites were found disease-specific and others matched also with other neurodegenerative diseases such as Alzheimer’s disease and amyotrophic lateral sclerosis. A more specific set of different system biomarkers should be studied for PD diagnosis or prognosis or both.

In vivo brain metabolomics in PD can be studied noninvasively by magnetic resonance spectroscopy (MRS) measured in specified small tissue volumes in the brain; this technique is useful for increasing insight into the pathophysiological mechanisms underlying PD and may produce biomarkers for metabolic dysfunctions reflecting irreversible neuronal damage (Davie 1998). This technique is based on the behaviour of specific nuclei within a magnetic field and on the principle that resonant frequencies depend on the chemical environment around the nuclei. Clinical studies mainly focused on metabolites containing hydrogen (H MRS), providing information about the status of high-energy phosphates, and those containing phosphorus (31P MRS), reflecting intracellular energy (Rango 2015). The extreme heterogeneity in these studies in number of enrolled patients and techniques used makes MRS, for the time being, only a future candidate in PD biomarker scenery (Fig. 4).

## Inflammation

Animal and human studies supported the role of inflammation in PD, due to the abnormal protein misfolding, which can activate a proinflammatory state involving both Th1 and Th2 responses (Mosley et al. 2012) and microglial activation (Qian and Flood 2008). This inflammatory activation depends also on interactions between aging, environmental risk factors and genetic factors. Dopaminergic drugs can modify the biological characteristics of T lymphatic cells dopamine receptors, by inhibiting the release of cytotoxic mediators involved in PD development. In their review illustrating immune dysregulation and inflammation response (Chen et al. 2016) Chen et al. identified several potential PD biomarkers: immunological genes (such as some human leukocyte antigen genes), PD-related genes (directly influencing α-synuclein accumulation), PD-related microRNAs (which modulate PD genes at a post-transcriptional level), specific antibodies (such as elongation factor 1-alpha-1 and poly (A) binding protein cytoplasmic-3), inflammatory factors (such as cytokines). Increased interleukin-6 and -8 levels indicate a risk of PD (Polivka et al. 2016); higher levels of soluble TNF receptor-1 were linked to early onset PD (Scalzo et al. 2010).

Among the various available PET radiotracers for imaging microglia, the most currently used is the 18 kDa translocator protein (TSPO), overexpressed on the outer mitochondrial membrane of the activated microglia, but usually poorly expressed in healthy brains (Gerhard 2016). Previous studies showed how microglia activation patterns differed in distinct parkinsonian syndromes according to how neuropathologic changes are distributed in these disorders, suggesting a role of a disease specific marker. Longitudinal studies are needed to estimate the association between biomarker evidence for microglial activation with and clinical disease progression in PD. Whether and if so how microglial activation relates to neurodegeneration in PD is an interesting question that deserves future research.

## Conclusion

Identifying a successful biomarker depends inevitably on fully understanding the pathophysiology underlying the disease. In PD, despite remarkable advances in our insight into the responsible mechanisms, the aetiology remains unknown. This drawback makes the search for diagnostic or preclinical biomarkers especially complex to achieve. Intense research efforts aim now to find multiple biomarkers to detect PD as early as possible and to distinguish it from other parkinsonisms. Although this evaluative review summarises research that takes the field ahead, given the abundant research it inevitably leaves some studies unmentioned. Given that the diagnosis of PD is still based on clinical findings, a single test seems unlikely to satisfy all the functions required by a reliable biomarker for PD. Current research now aims to strengthen the diagnosis of PD by combining various clinical or non-clinical biomarkers or both. A single biomarker could play one or multiple roles (preclinical, diagnostic, or prognostic) thus providing variable clinometric properties. A reliable putative biomarker should be reproducible and replicable and take into account possible confounding from sociodemographic or other factors (McGhee et al. 2013). A feature that makes discovering a biomarker an even more complex undertaking is the clinical heterogeneity in PD from the early preclinical to the more advanced disease stages. Aggregation of certain symptoms in specific clusters (expressed by different phenotypic PD traits) may reflect degeneration in distinct neural pathways (Di Battista et al. 2018), a pathophysiological change that accounts for different expression and also disease progression. The variability in findings from biomarker studies might reflect recruiting a non-uniform PD population. Heterogeneity in PD could imply the need to seek specific diagnostic biomarkers for variable PD traits, and consequently, different theragnostic markers. Research efforts to this aim may be very beneficial when research develops neuroprotective therapies to slow or halt the progression of the disease. Despite advances in our insight into the pathogenesis and pathophysiology underlying PD, today the neurologist has no 100% reliable clinical marker for use in clinical practice and still needs to rely on clinical expertise to diagnose PD. This is the most pressing problem on which biomarker researchers need to focus their efforts in the coming years.
